# Improvements in data completeness in health information systems reveal racial inequalities: longitudinal national data from hospital admissions in Brazil 2010–2022

**DOI:** 10.1186/s12939-024-02214-3

**Published:** 2024-07-18

**Authors:** Rony Coelho, Rudi Rocha, Thomas Hone

**Affiliations:** 1 Instituto de Estudos Para Políticas de Saúde, São Paulo, Brazil; 2 São Paulo School of Business Administration (FGV EAESP), São Paulo, Brazil; 3 Public Health Policy Evaluation Unit, School of Public Health, Imperial College London, London, England

**Keywords:** Race and ethnicity, Data completeness, Racial disparities, Hospital admissions, Social determinants on health

## Abstract

**Background:**

Race and ethnicity are important drivers of health inequalities worldwide. However, the recording of race/ethnicity in data systems is frequently insufficient, particularly in low- and middle-income countries. The aim of this study is to descriptively analyse trends in data completeness in race/color records in hospital admissions and the rates of hospitalizations by various causes for Blacks and Whites individuals.

**Methods:**

We conducted a longitudinal analysis, examining hospital admission data from Brazil’s Hospital Information System (SIH) between 2010 and 2022, and analysed trends in reporting completeness and racial inequalities. These hospitalization records were examined based on year, quarter, cause of admission (using International Classification of Diseases (ICD-10) codes), and race/color (categorized as Black, White, or missing). We examined the patterns in hospitalization rates and the prevalence of missing data over a period of time.

**Results:**

Over the study period, there was a notable improvement in data completeness regarding race/color in hospital admissions in Brazil. The proportion of missing values on race decreased from 34.7% in 2010 to 21.2% in 2020. As data completeness improved, racial inequalities in hospitalization rates became more evident – across several causes, including assaults, tuberculosis, hypertensive diseases, at-risk hospitalizations during pregnancy and motorcycle accidents.

**Conclusions:**

The study highlights the critical role of data quality in identifying and addressing racial health inequalities. Improved data completeness has revealed previously hidden inequalities in health records, emphasizing the need for comprehensive data collection to inform equitable health policies and interventions. Policymakers working in areas where socioeconomic data reporting (including on race and ethnicity) is suboptimal, should address data completeness to fully understand the scale of health inequalities.

## Introduction

Race and ethnicity are critical social determinants of health [1–4]. The coronavirus pandemic and high-profile events such as the death of George Floyd and the Black Lives Matter movement have further emphasized the large and unjust inequalities affecting populations experiencing racial discrimination [5]. Racial and ethnic inequalities are pervasive, yet in many countries there is a lack of data to examine these inequalities or, where data is collected by race and/or ethnicity, it is of poor quality [6]. This issue is particularly acute in low- and middle-income countries (LMICs) [7].

Without accurate measurement, differences in health outcomes and healthcare utilisation may remain undetected, inhibiting robust measurements of inequalities and targeted efforts to improve services and coverage. Disaggregating data by patient race/ethnicity is crucial for identifying and addressing racial/ethnic health inequalities [8]. Evidence suggests that the data quality may differ for different racial groups. In general terms, minority and more vulnerable groups tend to have worse completeness [9, 10]. Data collection for health research is influenced by individuals’ willingness and ability to provide data and the priorities of those involved in collecting and investing in the data. Data can mirror the inequalities and inequities present in society. Ethnic minority groups have lower participation rates in research due to reasons such as mistrust and fear of the medical establishment, stigma related to research participation, and intentional exclusion [11].

Brazil is important for evaluating racial health inequalities and providing new evidence beyond the extensive literature from high-income countries [12, 13]. In the 2022 Census, approximately 57% of the Brazilian population identified themselves as Black or Brown/mixed race [14]. The country has a long history of colonization and slavery, and significant racial health inequalities persist today. The Black population has lower access to health care and suffers discrimination in health care services [15–19]. For example, Brazilian children of Black and Brown mothers are 72% more likely to die from diarrhea than children of white mothers [20]. Black Brazilians in Rio de Janeiro are 3.7 times more likely to die from mental illness than White Brazilians with the same level of education [21]. Individuals of black race/ethnicity and other socioeconomically disadvantaged groups are more likely to have multimorbidity, which increases hospital admissions and mortality rates.

Brazil's national health service is publicly funded and provides free healthcare at the point of care through the Sistema Único de Saúde (SUS; Unified Health System) [22]. Over the last 30 years, there have been large increases in coverage and access to health services, leading to better overall health outcomes. Despite this, access to specialized care remains a major issue in the public health sector, with long wait times persisting. Approximately 71.5% of the Brazilian population relies on the publicly-funded SUS for healthcare. Although all Brazilians are covered by the SUS, 28.5% of the population (primarily those with higher incomes and living in large urban centers) are covered by private health insurance plans.

One of the fundamental principles of the SUS is to reduce health disparities. Brazil currently ranks as one of the most economically unequal countries in the world, with a Gini index of 48.9 in 2020 [23]. With a population of over 210 million and a country of continental dimensions, Brazil also faces significant internal regional inequalities. The majority of the population is composed of black and mixed-race individuals, groups that exhibit the worst socioeconomic indicators. White Brazilians consistently have an overrepresentation in higher income stratas [24], and have higher educational levels [25] and longer life expectancy [26]. Based on the 2022 Demographic Census, 57.2% (138.5 million) of the Brazilian population self-identify as Black/Brown, highlighting the substantial influence of African ancestry. Approximately 47.0% (113.1 million) of the population self-identify as Brown, whereas 10.2% (24.4 million) self-identify as Black. The White population accounts for 43.5% (104.2 million) of the total, while the Indigenous and Yellow (Asian) populations represent 0.6% (1.4 million) and 0.4% (1.0 million) respectively [14].

Data collection on race in the public health information systems started in 1996 in Brazil, beginning with birth and mortality records [27]. In 2008, racial data collection became mandatory for hospital admissions and outpatient records, but with the option to register the value as 'uninformed' or leave it blank. However, evidence indicates that incomplete data disproportionately affects Black or Brown/mixed race (known as pardo) individuals [28, 29]. One reason stems from the historically lower level of access to health services that Black and Pardo Brazilians have [22]. There have also been changes in the social perception of what it means to be “Black” over recent years in Brazil. Increased positive social perceptions of blackness, largely driven by public policies such as quotas in universities, have led to more people identifying as Black or Brown/mixed in a non-stigmatized way [30].

However, there remains a dearth of studies evaluating how data completeness is associated with the magnitude of racial/ethnic health inequalities [27], especially in LMICs. This study descriptively analyzes the completion of racial administrative data in hospital admissions in Brazil, and explores trends over time in the completeness of the race/color on hospital records and the rates of hospitalizations for various causes of hospitalization for whites and blacks. This is a crucial evidence gap for convincing policymakers and administrators to act on the issue of racial/ethnic health inequalities. We examine the completeness of racial data on hospital admissions in Brazil for a range of causes between 2010 and 2022 and document how measures of racial inequalities have evolved as reporting improves.

## Methods

### Study design

This study is a longitudinal analysis of publicly-funded hospitalization rates in Brazil from 2010–2022 and its correlation with data completeness. We investigated patterns in hospitalization rates from January 2012 to December 2022 and the proportion of missing data over time.

### Data sources

Data from the Hospital Information System in Brazil (SIH) were obtained (https://datasus.saude.gov.br/) from January 2010 to June 2022. This data includes 145,554,343 hospital admissions records for all publicly-funded admissions.

The Hospital Information System of DataSUS (SIH/SUS) is the main source of data on hospital admissions within the Unified Health System (SUS) in Brazil. Information collected includes diagnoses, procedures, length of stay, and patients' personal data. These data are generated as part of the administrative activity of the State, specifically in the provision of health services by SUS and the receipt of financial counterpart. All health units that serve patients through SUS must report admission data to SIH/SUS monthly, making them mandatory administrative records. These data are made publicly available in an anonymized format. Thus, SIH/SUS has also become a crucial source of data for epidemiological and public health analyses.

According to Brazilian legislation [31], the collection of the racial category and filling out the race/ethnicity field are required in healthcare services based on the user's self-declaration criteria, as per Brazilian Institute of Geography and Statistics (IBGE) standards. In the case of newborns, deaths, or when the user is unable to self-declare, family members or guardians will declare their race or ethnicity. If there is no responsible party, healthcare professionals will complete the race/ethnicity field during the service.

The official racial/skin colour categories in Brazil include Black, Brown, White, Indigenous, and Yellow (Asian) [32].

Individuals categorize themselves into racial groups based on skin tone, hair texture, facial structure, and cultural and ethnic traits [33].

We aggregated individual admission records by year and quarter, primary cause of admission (by International Classification of Diseases (ICD-10) codes), and by race/color. We grouped hospital admissions into three groups: Blacks, Whites, and missing. Therefore, the Black group includes all Black and Mixed race individuals (Brown or Pardo)—given the historical degree of racial mixing, and as frequently done in the scientific literature [34, 35]. Asians and Indigenous peoples were not analyzed due to low numbers and also, especially related to indigenous people, given unique characteristics related to living conditions and specificities related to access to health services and other indigenous policies.

We use data from the Continuous National Household Sample Survey (PNAD-Contínua) of the IBGE for population estimates for the period of 2012–2022. This survey is carried out continuously throughout the year, collecting data on the population, employment, income, education, and other socio-economic characteristics of Brazilian households. It is through the PNAD-Contínua that, for example, the official unemployment rate in the country is calculated. It provides a comprehensive view of the country's socio-economic characteristics, allowing for the tracking of trends over time and the comparison of different population groups with quarterly data. We obtained estimates of the population by race/color by each quarter for the entire period as the denominator for rates. SIH data is collected on a monthly basis, while PNAD-Contínua are collected quarterly. Month/quarter compatibility was used to calculate the rates.

We considered the following causes that are recognized for their significant racial inequalities in Brazil [15–19, 36]: motorcycle accidents (ICD-10 V20-V29); assaults (ICD-10 X92-Y09), tuberculosis (ICD-10 A15-A19); hypertensive diseases (ICD-10 I10-I15); at-risk hospitalizations during pregnancy (known as “near miss” [37], see Appendix 1).

The data from the Hospital Information System (SIH) and IBGE are publicly accessible and anonymized. Ethical principles of transparency, privacy, confidentiality, and protection of individual rights of participants are therefore respected.

### Analysis

We first estimated hospitalization rates for Black and White individuals per 10,000 inhabitants (of respective race/color), for each group of causes of admission, using hospitalization data from SIH [38] and national population data from PNAD-Continua as a population denominator for calculating rates. Secondly, we calculated the share of admissions for each category (Whites, Blacks and missing values), summing up 100% for each quarter-year. Thirdly, we computed the ratio of hospitalization rates between Blacks and Whites to quantify the inequalities in hospitalization rates between Blacks and Whites over time. Therefore, we are left with an indication of the rate ratio between Black and White, in which if there is a value equal to 1, it means that there are no racial differences. A value below 1 indicates inequalities in the disadvantage experienced by the White population, while a value above 1 indicates inequalities in the disadvantage experienced by the Black population.

Time series were plotted for hospital admissions of all causes and by groups of causes using 12-month moving averages.

## Results

In 2010, over a third (34.7%) of publicly-funded hospital admissions in Brazil had missing values for race/color, yet by 2020 this was 21.2%. There was a consistent increase in reporting over time for all causes of hospital admission.

**Fig. 1:**
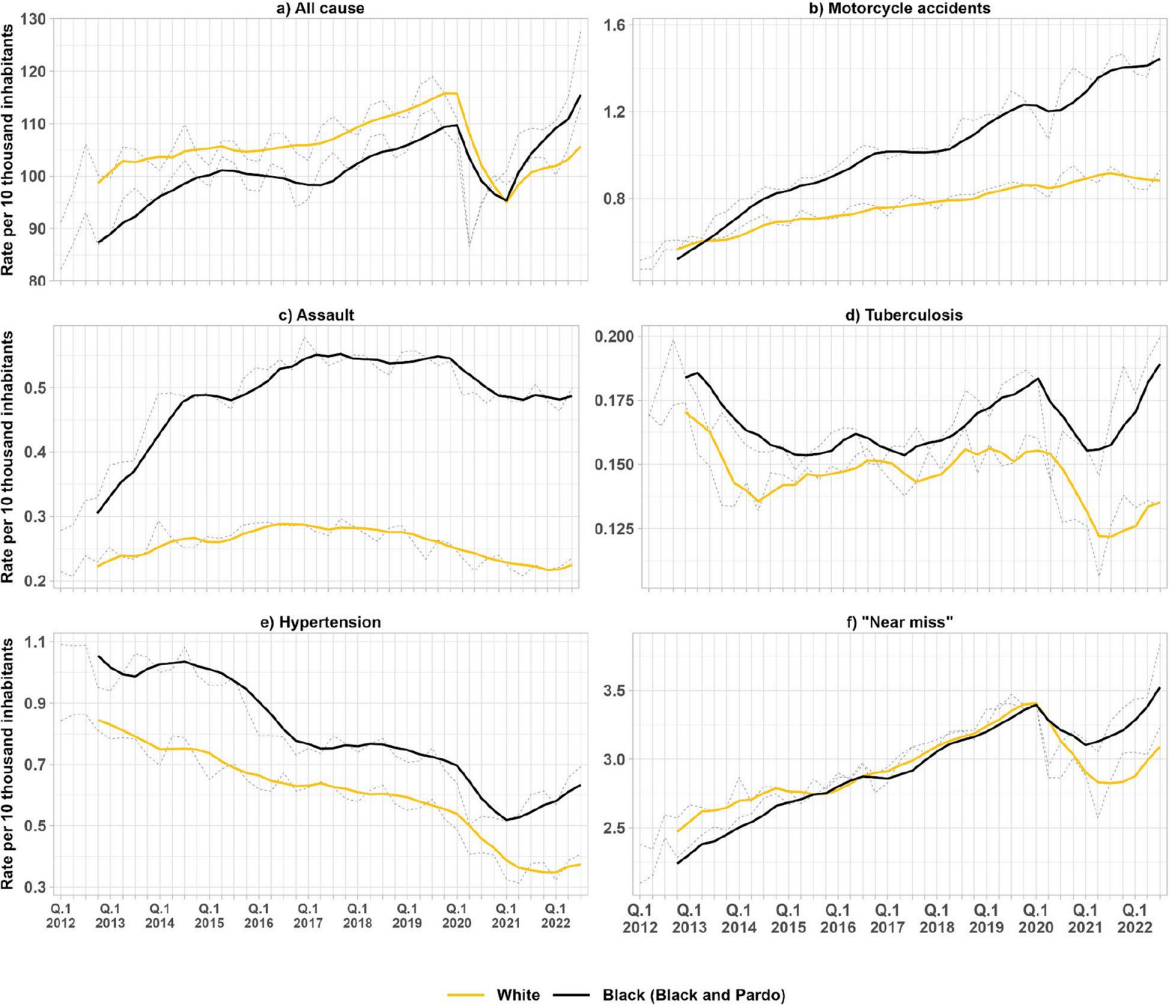
Trends of rates of hospital admission in Brazil by race/color. Note: the figure shows the evolution of hospitalization rates for Blacks and Whites in Brazil. The data is from the SIH, and the rates were calculated per 10,000 inhabitants. The grey dashed lines show the quarterly rates whose population data comes from the PNAD-C. The colored lines show the moving averages every four quarters

For all causes of hospital admissions (Fig. 1), White Brazilians have had higher rates of hospital admission before the COVID-19 pandemic, although the rate for Black Brazilians has increased faster over time than for White Brazilians and has recently overtaken them. In the first quarter of 2012, the hospital admission rate for Whites was 91.2 per 10,000, and for Blacks, it was 82.2. In the first quarter of 2022, the rate was approximately 110 for Blacks and 100 for Whites.

Hospital admission rates, trends, and inequalities vary for cause. Black Brazilians experience higher rates of admission for assault, tuberculosis, and hypertension than White Brazilians. For motorcycle accidents, although the rates for Blacks and Whites were similar in 2012 and 2013, they have diverged since then, with admissions for Black Brazilians increasing at a greater rate than for the White population.

**Fig. 2:**
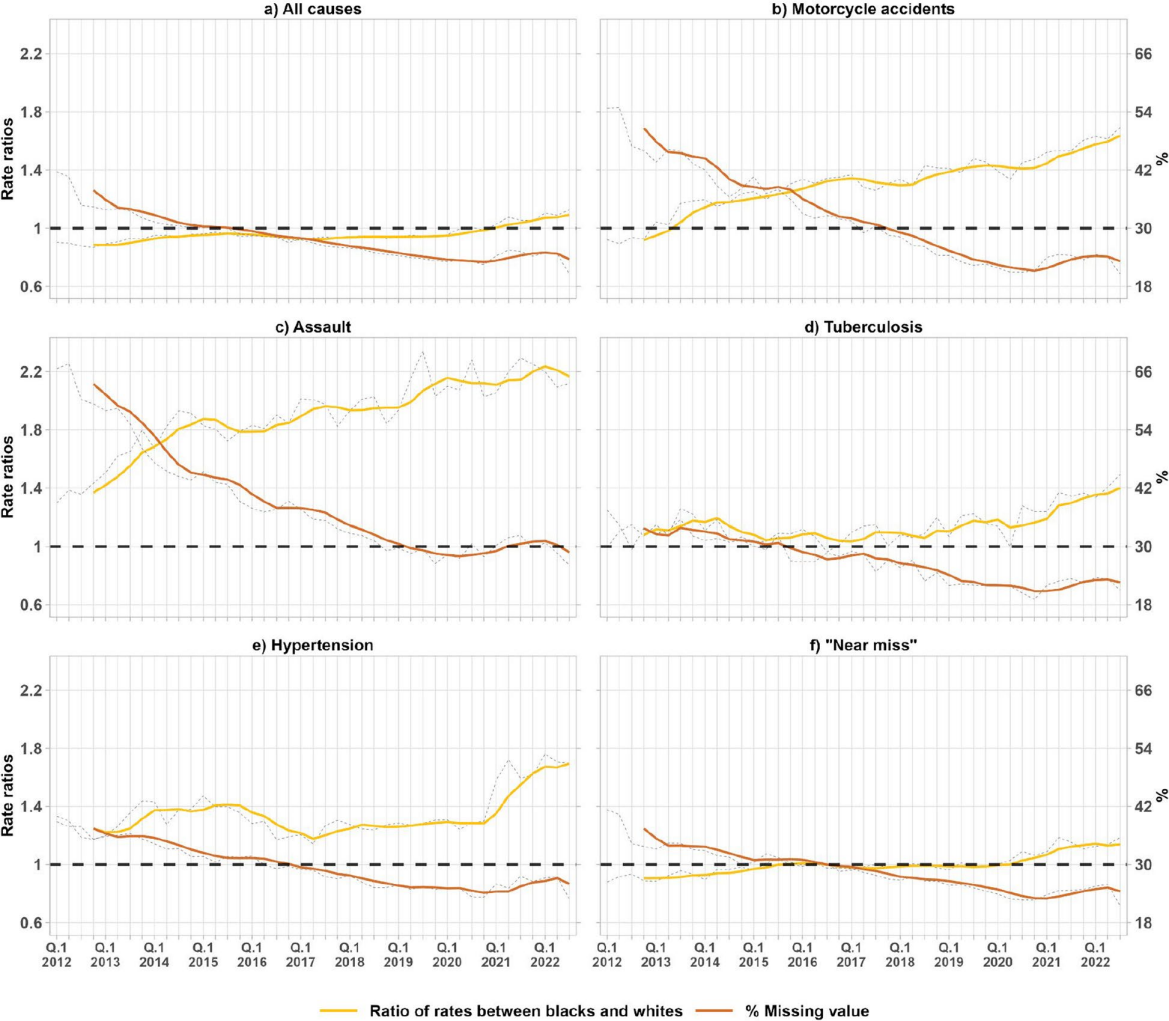
Trends in missing values proportion and hospital admission ratio for Blacks and Whites. Note: The figure shows, on the left axis and with the orange line, the evolution of the ratio of hospitalization rates between Blacks and Whites. On the right-hand axis and the yellow line it shows the evolution of the proportion of missing data. The dashed grey lines show the quarterly rates whose population data comes from the PNAD-C. In the case of the proportion (right axis), the dashed grey lines show the quarterly proportions. The colored lines show the moving averages every four quarters

Exploring the relationship between racial inequalities in admissions (measured by rate ratios) and the proportion of admissions with missing data reveals an inverse relationship between racial inequalities and missing data (Fig. 2, Appendix Sections 2 and 3). As data completeness for admissions has improved over time, racial inequalities between Black and White Brazilians become more evident. For example, for motorcycle accidents, 46.2% of admissions had missing race/ethnicity data in 2011, which decreased to 23% in 2022. However, the rate ratio between Black and White admissions was 0.92 in the first quarter of 2012, increasing to 1.53 in the first quarter of 2021. A similar pattern was identified for other causes. By 2022, when missing data had been substantially reduced, the hospitalization rate for Black Brazilians was greater than for White Brazilians for all causes studied.

## Discussion

This study documents that racial inequalities in hospital admission rates between Black and White Brazilians have become more pronounced over time as the completeness of racial records improves.

Throughout the analyzed period, we observed a trend of improvement in the completeness of racial data in hospital records. In 2010 about one-third of public admissions had missing or incomplete information on race/color. That figure decreased by approximately 38.9% by 2020. This improvement in data accuracy also reveals inequalities in admission rates for specific causes — such as for assaults, tuberculosis, and hypertension, where Black Brazilians consistently exhibit higher rates compared to Whites (Figs. 1 and 2). Improvement in data completeness is crucial for a more precise and comprehensive understanding of racial health inequalities, and underscores the importance of data collection and recording systems to support more equitable and effective health policies.

Multiple reasons underlie these findings. Factors such as access to healthcare, underlying health conditions, health system resourcing, and socioeconomic inequalities likely explain these findings. Literature from high-income countries indicates that minority and socially disadvantaged groups are less likely to have their data recorded [8–10, 39]. For example, evidence from the US on HealthCare.gov enrollment data showed that non reporters were disproportionately Black or Hispanic [40]. In Brazil [41], a study of a university hospital found that 83% of the valid records for race were for White people, 11% for Mixed race, and only 5% for Black individuals. Potential explanations from this study identified a need for more specific training for health professionals for completing the race/color questionnaire, and that staff were afraid of the reaction of patients when asked to self-declare their race/ethnicity.

Our results show that the gradual reduction of missing data on race/color in hospital records allows for a more accurate representation of existing racial inequalities. Evidently, multiple factors contribute to this trend. One such factor may plausibly be the greater proportion of Black Brazilians historically facing incomplete or inadequately documented admission records compared to their White counterparts [42, 43]. Another explanation for the Brazilian case may be increased access to health services in recent decades [43]—particularly in more deprived areas with greater racial disparities in health and a larger proportion of black people [22]. The Unified Health System (SUS) expansion has increased the availability of primary care, increasing the number of people covered by the system [43]. More recently, evaluations have shown that the difference in distance to health services between whites and blacks has diminished [44]. Expanding primary care may have also facilitated increased access to hospitals, increasing access and utilization for Black Brazilians.

A recent national survey shows that the proportion of people declaring themselves Black has also increased over the last decade [37], demonstrating a shift in the social perception of the racial matters in Brazil. The increased visibility and representation of black individuals in media, politics, and public spheres have influenced society's perception of the black community [45, 46]. Furthermore, the adoption of public policies like racial quotas in universities and public organizations has led to a rise in the representation of black individuals in areas typically occupied by white individuals [30]. These actions are likely correlated with an increased number of individuals identifying as black, and potentially explain part of the larger increases in hospital admission rates for Blacks individuals than for whites over time.

There are limitations with this study. Non-hospital health outcomes are not considered, and only publicly-funded admissions and selected causes are analyzed. By focusing solely on hospital admissions and excluding other health data such as birth records, mortality, and outpatient data, the study only provides a partial picture in the evolution of the measurement of racial inequalities. Limiting the analysis to publicly-funded admissions and selected causes cover only certain segments of the population or health conditions. Privately-funded admissions may reflect different patterns of healthcare utilization and may be associated with different health outcomes compared to publicly-funded admissions. However, the vast majority of Black Brazilians rely exclusively on the SUS and therefore are covered by our analysis. One explanation of the inequalities between black and white Brazilians may be due to a higher usage of the private sector by white Brazilians [47], although private insurance coverage has remained remarkably stable over time. On the other hand, focusing on selected causes may overlook disparities in other health conditions that disproportionately affect certain racial groups. Underlying causes of the observed trends are not explored and causality cannot be established between improvements in data completeness and the emergence of racial inequalities. Statistical tests were also not undertaken to evaluate these inequalities and trends. Furthermore, the socioeconomic factors which are associated with skin color/race, admission rates, and likelihood of missing data are not taken into account.

The production of epidemiological data disaggregated by race/color is vital for understanding the specific health needs of different racial/ethnic groups, developing more effective and equitable health policies, and delivering services that can address the needs of the communities served. This includes policies specifically addressing racial/ethnic health inequalities. In Brazil, the monitoring and evaluation of the National Policy for the Integral Health of the Black Population (NPIBP) depends on data on racial/ethnic indicators. The NPIBP aims to reduce racial inequalities in health and make health professionals and society aware of these inequalities [36]. Awareness campaigns regarding the importance of race/color data collection are also important. Accurate data is essential for the effective implementation of health policies, and in our context this is particularly relevant for the implementation of the NPIBP. Awareness campaigns should include training healthcare professionals to accurately collect data, which by its turn is instrumental to identify and address racial health disparities.

## Conclusions

The study highlights the importance of data completeness in identifying and understanding racial inequalities in health, and emphasizes how in recent years black Brazilians experience higher hospital admissions rates than white Brazilians. Comprehensive and accurate data is vital for informed equitable policies and interventions, and addressing unjust racial/ethnic health inequalities.

## Figures and Tables

**Fig. 3:**
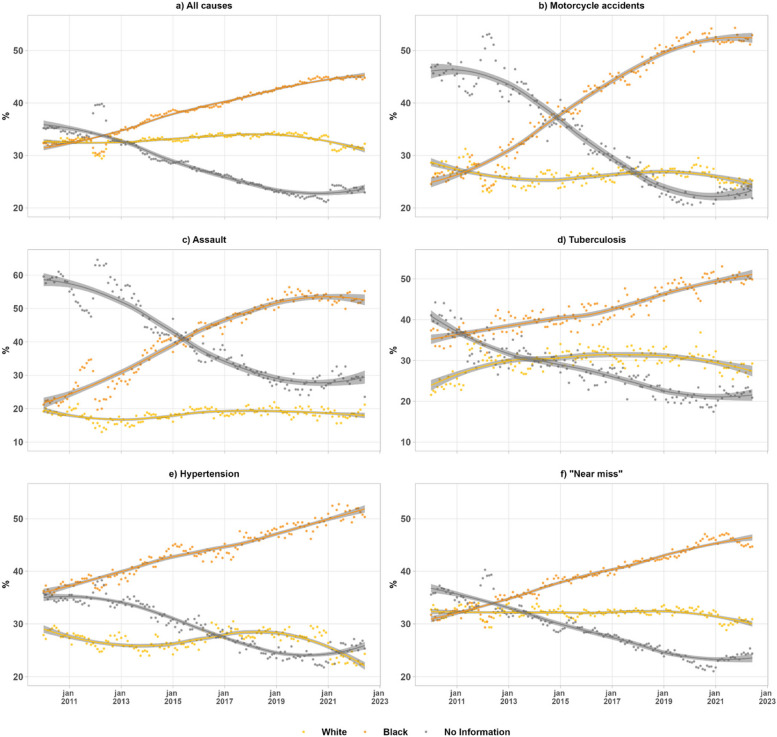
Trends in the proportions of Blacks and Whites individuals and missing values. Note: Each point on the graph represents the percentage of each of the categories (Black, White and No information) in each month so that their sum, in each month, adds up to 100%. Trend lines and confidence intervals (95%)—gray region—were calculated using the standard LOESS (Local Regression) smoothing method, a non-parametric technique that fits a smooth curve to the data, considering a local region around each point. LOESS is particularly useful for capturing complex patterns in data because it does not make hard assumptions about the form of the relationship between variables. This method, by default, is used by the geom_smooth function from the ggplot package in the R program. The information here is the same as that present in Fig. 2, in which we present the ratio of the proportion between blacks and whites (left axis) and the missing percentage (right axis)

## Data Availability

All data used in this study are publicly available at https://datasus.saude.gov.br/ and https://www.ibge.gov.br/. The authors will also make the data available upon request.

## Appendix 1

### CIDs of Near miss

For “Near miss”, we consider the following CIDs, as described in the literature [37]: A021, A227, A267, A327, A40, A400, A401, A402, A403, A408, A409, A41, A410, A411, A412, A413, A414, A415, A418, A419, A427, A548, B377, D62, K350, K359, K650, K658, K659, M869, N700, N709, N710, N733, N735, O030, O031, O035, O036, O040, O041, O045, O046, O050, O051, O055, O056, O060, O061, O065, O066, O070, O071, O075, O076, O080, O081, O082, O083, O11, O140, O141, O149, O15, O150, O151, O152, O159, O411, O441, O450, O458, O459, O46, O460, O468, O469, O53, O710, O711, O720, O721, O722, O753, O85, O860, O868, O883, O900.

## Appendix 3

**Table 1:** Rate ratios, percentage of missing values and its corresponding rollmeans for various causes and health conditions

Table												
	All cause				Motorcycle accidents				Assault			
Quarters	Rate ratios	Rollmean of Rate ratios	% of missing values	Rollmean of the % of missing values	Rate ratios	Rollmean of Rate ratios	% of missing values	Rollmean of the % of missing values	Rate ratios	Rollmean of Rate ratios	% of missing values	Rollmean of the % of missing values
2012.1	0.90	-	0.40	-	0.92	-	0.52	-	1.30	-	0.63	-
2012.2	0.90	-	0.39	-	0.89	-	0.52	-	1.38	-	0.64	-
2012.3	0.88	-	0.33	-	0.94	-	0.45	-	1.36	-	0.57	-
2012.4	0.87	0.89	0.33	0.36	0.92	0.92	0.44	0.48	1.43	1.37	0.57	0.60
2013.1	0.89	0.88	0.32	0.34	1.04	0.95	0.41	0.46	1.51	1.42	0.55	0.58
2013.2	0.91	0.89	0.32	0.33	1.02	0.98	0.44	0.44	1.62	1.48	0.56	0.56
2013.3	0.93	0.90	0.32	0.32	1.17	1.04	0.44	0.43	1.65	1.55	0.53	0.55
2013.4	0.93	0.91	0.31	0.32	1.19	1.11	0.41	0.43	1.80	1.64	0.48	0.53
2014.1	0.95	0.93	0.30	0.31	1.20	1.14	0.40	0.42	1.67	1.68	0.45	0.50
2014.2	0.95	0.94	0.29	0.30	1.15	1.18	0.37	0.40	1.83	1.74	0.43	0.47
2014.3	0.94	0.94	0.29	0.30	1.18	1.18	0.35	0.38	1.93	1.81	0.42	0.45
2014.4	0.96	0.95	0.29	0.29	1.24	1.19	0.36	0.37	1.92	1.84	0.42	0.43
2015.1	0.96	0.95	0.29	0.29	1.25	1.20	0.39	0.37	1.83	1.88	0.43	0.43
2015.2	0.97	0.96	0.29	0.29	1.20	1.22	0.35	0.36	1.80	1.87	0.41	0.42
2015.3	0.96	0.96	0.29	0.29	1.24	1.23	0.36	0.37	1.72	1.82	0.41	0.42
2015.4	0.94	0.96	0.27	0.28	1.30	1.25	0.34	0.36	1.79	1.79	0.37	0.41
2016.1	0.95	0.96	0.27	0.28	1.34	1.27	0.31	0.34	1.83	1.79	0.36	0.39
2016.2	0.95	0.95	0.27	0.28	1.31	1.30	0.30	0.33	1.81	1.79	0.35	0.37
2016.3	0.94	0.94	0.27	0.27	1.34	1.32	0.31	0.32	1.90	1.83	0.36	0.36
2016.4	0.90	0.93	0.26	0.27	1.35	1.33	0.30	0.31	1.85	1.85	0.37	0.36
2017.1	0.93	0.93	0.26	0.27	1.37	1.34	0.30	0.30	2.01	1.89	0.36	0.36
2017.2	0.93	0.92	0.26	0.26	1.28	1.34	0.28	0.30	2.01	1.94	0.34	0.36
2017.3	0.94	0.93	0.25	0.26	1.26	1.31	0.29	0.29	1.98	1.96	0.34	0.35
2017.4	0.93	0.93	0.25	0.25	1.31	1.30	0.27	0.28	1.83	1.96	0.32	0.34
2018.1	0.94	0.94	0.25	0.25	1.34	1.30	0.27	0.28	1.93	1.93	0.31	0.33
2018.2	0.95	0.94	0.24	0.25	1.29	1.30	0.25	0.27	2.01	1.94	0.31	0.32
2018.3	0.94	0.94	0.24	0.24	1.43	1.34	0.25	0.26	2.03	1.95	0.30	0.31
2018.4	0.93	0.94	0.23	0.24	1.41	1.37	0.23	0.25	1.84	1.95	0.28	0.30
2019.1	0.94	0.94	0.23	0.24	1.41	1.39	0.23	0.24	1.94	1.95	0.28	0.29
2019.2	0.95	0.94	0.23	0.23	1.38	1.41	0.22	0.23	2.16	1.99	0.27	0.28
2019.3	0.95	0.94	0.23	0.23	1.48	1.42	0.21	0.22	2.34	2.07	0.28	0.28
2019.4	0.94	0.94	0.22	0.23	1.46	1.43	0.22	0.22	2.03	2.12	0.25	0.27
2020.1	0.96	0.95	0.22	0.22	1.39	1.43	0.21	0.21	2.10	2.16	0.27	0.27
2020.2	1.00	0.96	0.22	0.22	1.34	1.42	0.20	0.21	2.07	2.14	0.26	0.27
2020.3	1.01	0.97	0.22	0.22	1.45	1.41	0.20	0.21	2.28	2.12	0.29	0.27
2020.4	0.98	0.99	0.21	0.22	1.47	1.41	0.20	0.20	2.03	2.12	0.27	0.27
2021.1	1.03	1.00	0.23	0.22	1.52	1.45	0.23	0.21	2.05	2.11	0.29	0.28
2021.2	1.08	1.02	0.24	0.23	1.53	1.49	0.23	0.22	2.20	2.14	0.30	0.29
2021.3	1.05	1.04	0.24	0.23	1.53	1.51	0.23	0.22	2.29	2.14	0.31	0.29
2021.4	1.05	1.05	0.23	0.24	1.61	1.55	0.22	0.23	2.25	2.20	0.28	0.30
2022.1	1.10	1.07	0.24	0.24	1.63	1.58	0.23	0.23	2.20	2.24	0.29	0.30
2022.2	1.09	1.07	0.24	0.24	1.61	1.60	0.23	0.23	2.09	2.21	0.27	0.29

**Table **												
	**Tuberculosis**				**Hypertensions**				**Near Miss**			
**Quarters**	**Rate ratios**	**Rollmean of Rate ratios**	**% of missing values**	**Rollmean of the % of missing values**	**Rate ratios**	**Rollmean of Rate ratios**	**% of missing values**	**Rollmean of the % of missing values**	**Rate ratios**	**Rollmean of Rate ratios**	**% of missing values**	**Rollmean of the % of missing values**
2012.1	1.00	-	0.36	-	1.30	-	0.38	-	0.88	-	0.39	-
2012.2	1.10	-	0.33	-	1.26	-	0.37	-	0.92	-	0.38	-
2012.3	1.15	-	0.28	-	1.26	-	0.34	-	0.94	-	0.33	-
2012.4	1.07	1.08	0.32	0.32	1.17	1.25	0.34	0.36	0.89	0.91	0.32	0.36
2013.1	1.15	1.12	0.31	0.31	1.20	1.22	0.34	0.35	0.89	0.91	0.32	0.34
2013.2	1.08	1.11	0.32	0.31	1.27	1.22	0.34	0.34	0.92	0.91	0.33	0.32
2013.3	1.26	1.14	0.34	0.32	1.36	1.25	0.35	0.34	0.96	0.91	0.33	0.32
2013.4	1.22	1.18	0.31	0.32	1.44	1.31	0.34	0.34	0.94	0.93	0.32	0.32
2014.1	1.11	1.17	0.30	0.31	1.43	1.37	0.33	0.34	0.90	0.93	0.31	0.32
2014.2	1.18	1.19	0.30	0.31	1.28	1.38	0.32	0.33	0.97	0.94	0.30	0.31
2014.3	1.04	1.14	0.29	0.30	1.38	1.38	0.32	0.32	0.97	0.94	0.30	0.31
2014.4	1.07	1.10	0.30	0.30	1.38	1.37	0.30	0.32	0.98	0.95	0.29	0.30
2015.1	1.03	1.08	0.29	0.30	1.47	1.38	0.30	0.31	0.96	0.97	0.29	0.29
2015.2	1.04	1.04	0.28	0.29	1.40	1.41	0.29	0.30	1.01	0.98	0.31	0.30
2015.3	1.09	1.06	0.31	0.29	1.40	1.41	0.30	0.30	1.04	1.00	0.30	0.30
2015.4	1.09	1.06	0.26	0.28	1.36	1.41	0.30	0.30	1.00	1.00	0.29	0.30
2016.1	1.12	1.08	0.26	0.27	1.28	1.36	0.30	0.30	0.99	1.01	0.28	0.30
2016.2	1.07	1.09	0.26	0.27	1.30	1.33	0.29	0.30	1.01	1.01	0.29	0.29
2016.3	0.96	1.06	0.27	0.26	1.17	1.28	0.28	0.29	1.01	1.00	0.28	0.29
2016.4	1.01	1.04	0.27	0.26	1.19	1.24	0.28	0.29	0.95	0.99	0.28	0.28
2017.1	1.10	1.03	0.28	0.27	1.21	1.22	0.28	0.28	0.96	0.98	0.28	0.28
2017.2	1.14	1.05	0.27	0.27	1.14	1.18	0.27	0.28	0.99	0.98	0.27	0.28
2017.3	1.15	1.10	0.24	0.26	1.27	1.20	0.26	0.27	1.00	0.98	0.26	0.27
2017.4	1.00	1.10	0.26	0.26	1.30	1.23	0.26	0.27	0.98	0.98	0.26	0.27
2018.1	1.08	1.09	0.24	0.25	1.28	1.25	0.26	0.26	0.99	0.99	0.26	0.26
2018.2	1.06	1.07	0.26	0.25	1.25	1.27	0.25	0.26	1.00	0.99	0.26	0.26
2018.3	1.09	1.06	0.22	0.25	1.24	1.27	0.24	0.25	1.00	0.99	0.25	0.26
2018.4	1.20	1.11	0.23	0.24	1.27	1.26	0.24	0.25	0.98	0.99	0.25	0.26
2019.1	1.07	1.10	0.21	0.23	1.29	1.26	0.25	0.24	0.97	0.99	0.25	0.25
2019.2	1.21	1.14	0.21	0.22	1.27	1.27	0.24	0.24	1.01	0.99	0.25	0.25
2019.3	1.22	1.18	0.21	0.22	1.28	1.28	0.24	0.24	0.98	0.98	0.24	0.25
2019.4	1.16	1.17	0.21	0.21	1.31	1.29	0.24	0.24	0.99	0.99	0.23	0.24
2020.1	1.14	1.18	0.21	0.21	1.31	1.29	0.24	0.24	1.00	1.00	0.23	0.24
2020.2	1.00	1.13	0.21	0.21	1.24	1.29	0.24	0.24	1.03	1.00	0.22	0.23
2020.3	1.28	1.14	0.19	0.20	1.29	1.29	0.22	0.23	1.09	1.03	0.22	0.22
2020.4	1.24	1.16	0.18	0.20	1.30	1.28	0.22	0.23	1.06	1.05	0.22	0.22
2021.1	1.24	1.19	0.21	0.20	1.58	1.35	0.25	0.23	1.09	1.07	0.23	0.22
2021.2	1.37	1.28	0.22	0.20	1.72	1.47	0.24	0.23	1.19	1.11	0.23	0.22
2021.3	1.34	1.30	0.22	0.21	1.59	1.55	0.26	0.24	1.15	1.12	0.24	0.23
2021.4	1.37	1.33	0.21	0.22	1.62	1.63	0.25	0.25	1.11	1.13	0.24	0.23
2022.1	1.34	1.35	0.22	0.22	1.76	1.67	0.26	0.25	1.13	1.14	0.24	0.24
2022.2	1.41	1.36	0.22	0.22	1.71	1.67	0.26	0.26	1.14	1.13	0.25	0.24

## Abbreviations

SIH: Hospital Information System
ICD-10: International Classification of Diseases
PNAD-Continua: Continuous National Household Sample Survey
LMICs: Low- and Middle-income countries
IBGE: Brazilian Institute of Geography and Statistics
